# Disrupting SARS-CoV-2 Spike–ACE2 Interactions via Glycosaminoglycans in a Pseudoviral Study of Heparan Sulfate and Enoxaparin

**DOI:** 10.3390/biom15070931

**Published:** 2025-06-26

**Authors:** Virginia Fuochi, Salvatore Furnari, Filippo Drago, Pio Maria Furneri

**Affiliations:** Department of Biomedical and Biotechnological Sciences (Biometec), University of Catania, 95123 Catania, Italy; salvatore.furnari@phd.unict.it (S.F.); filippo.drago@unict.it (F.D.)

**Keywords:** SARS-CoV-2, spike protein, ACE2 receptor, heparan sulfate, enoxaparin, viral entry inhibition, glycosaminoglycans

## Abstract

Background: The COVID-19 (coronavirus disease 19) pandemic has underscored the urgent need for effective antiviral agents targeting viral entry mechanisms. This study investigated the inhibitory effects of heparan sulfate (HS) and enoxaparin (EX) on the interaction between the severe acute respiratory syndrome coronavirus 2 (SARS-CoV-2) spike protein and the angiotensin-converting enzyme 2 (ACE2) receptor. Methods: A pseudovirus model was employed to evaluate the efficacy of HS and EX under different treatment strategies: pre-treatment of host cells, pre-treatment of the viral particles, and simultaneous co-treatment. Results: Both compounds significantly inhibited viral entry. EX exhibited a dose-dependent effect under all treatment conditions. In cell pre-treatment, EX achieved the highest levels of inhibition, whereas HS demonstrated consistent inhibitory activity that was largely concentration-independent. Viral pre-treatment revealed that both compounds effectively reduced infectivity by interfering directly with viral particles. In the co-treatment experiments, HS demonstrated superior inhibitory activity at lower concentrations compared to EX. Conclusions: The results suggested that HS and EX inhibit SARS-CoV-2 entry via distinct mechanisms. HS likely acts via competitive inhibition at the host cell surface, while EX may bind directly to the spike protein, thereby preventing engagement with the ACE2 receptor. These findings highlight the therapeutic potential of HS and EX as entry inhibitors targeting the early stages of SARS-CoV-2 infection. Further studies are warranted to evaluate their efficacy against emerging variants and in vivo models.

## 1. Introduction

Global efforts to address the impact of SARS-CoV-2 have emphasized both preventive and therapeutic approaches. While vaccines significantly reduced severe disease outcomes, the identification of effective antiviral agents remained a priority, particularly for limiting viral entry and replication in infected individuals. Previous findings indicated that SARS-CoV-2 entry could be effectively hindered by compounds targeting its spike protein and key host factors. Indeed, the potential of glycosaminoglycans (GAGs), specifically heparan sulfate (HS) and enoxaparin (EX), was demonstrated in inhibiting SARS-CoV-2 infection by disrupting the interaction between the viral spike protein and the ACE2 receptor on host cells [1,2]. Moreover, unfractionated heparin at clinically relevant concentrations demonstrated a pronounced capacity to reduce the infectivity of live wild-type SARS-CoV-2, underscoring its potential therapeutic utility [3]. Furthermore, it was conclusively established that viral entry depended not only on ACE2 but also on host cell heparan sulfate, reinforcing the rationale for targeting these glycosaminoglycan-dependent interactions [4]. Despite the progress made with vaccines, the emergence of new variants with enhanced transmissibility and immune evasion capabilities underscored the need for alternative or supplementary therapeutic options. Antiviral agents capable of targeting highly conserved viral processes, such as host cell attachment, may help mitigate the limitations posed by vaccine escape variants. In this context, HS and EX have emerged as promising candidates, as their mechanisms are not dependent on the specific mutational landscape of the spike protein [2]. This independence from sequence variability renders GAG-based strategies particularly appealing for combating emerging variants that may partially evade vaccine-induced immunity [5].

These findings laid the groundwork for the therapeutic application of GAGs, yet essential questions about their mechanisms of action and the conditions under which they perform optimally require further investigation. The interaction of HS and EX with the wild-type SARS-CoV-2 spike protein forms the basis for their antiviral activity. Recent work confirmed that HSPGs are important cofactors in SARS-CoV-2 entry and that their engagement is essential for viral internalization [6].

HS, a major GAG component of the cellular glycocalyx, plays a significant role in the early phase of viral attachment [7], enhancing the local concentration of the virus at the cell surface. This process preceded binding to the ACE2 receptor and presented an attractive target for therapeutic intervention. Similarly, EX, a low-molecular-weight heparin derived from heparin, demonstrated potential for interfering with this sequence [8]. Recent studies have also highlighted the multifaceted nature of EX, which includes potential anticoagulant and anti-inflammatory properties. These attributes could confer dual benefits by not only reducing viral load but also alleviating the systemic inflammation observed in severe COVID-19 cases. Exploring such pleiotropic effects in the context of SARS-CoV-2 infection has expanded its therapeutic appeal [1]. However, the conditions under which these compounds are most effective—whether through co-treatment with the virus, preconditioning of cells, or pre-treatment of the virus—have remained insufficiently explored. This study built upon prior findings by investigating the antiviral properties of HS and EX against wild-type SARS-CoV-2 under different experimental conditions [8]. Specifically, it evaluated their efficacy in three treatment scenarios: direct co-treatment of the virus and cells, pre-treatment of cells, and pre-treatment of the virus. These approaches helped clarify the circumstances under which their inhibitory effects are most pronounced and provided a better understanding of their mechanisms of action. Furthermore, while previous research focused primarily on antiviral efficacy, this study also examined the cytotoxic profiles of HS and EX to ensure their safety at therapeutic concentrations. By combining these evaluations, the study offered a more comprehensive assessment of their potential as antiviral agents against SARS-CoV-2 [7,9]. By refining the experimental conditions and elucidating the mechanisms through which HS and EX act, this research contributed to advancing their therapeutic application in preventing and managing viral infections. The findings aimed to support their development as targeted interventions for combating SARS-CoV-2.

Compared to existing studies on the inhibition of SARS-CoV-2 entry by glycosaminoglycans, this investigation employed a combinatorial approach using reduced concentrations of HS and EX, intended to simulate conditions suitable for intranasal delivery. A diversified treatment design was implemented, including cell pre-treatment, virus pre-treatment, and simultaneous exposure, allowing for the dissection of distinct antiviral mechanisms associated with each compound. In contrast to previous reports that typically focused on a single experimental setting or dose range, this multi-condition strategy provided a more comprehensive and clinically relevant evaluation of glycosaminoglycan-mediated inhibition of viral entry.

## 2. Materials and Methods

### 2.1. Chemicals, Cells, and Virus Models

HS and EX were supplied by Techdow Pharma S.r.l (Assago Milanofiori, 20090, Milan, Italy). Lung epithelial A549 cells (CCL-185™) were procured from the American Type Culture Collection (ATCC, Manassas, VA, USA). These cells were cultivated in F12K medium supplemented with 10% fetal bovine serum (FBS), 2 mM L-glutamine, and antibiotics (100 U/mL penicillin–streptomycin mix). The cultures were maintained at 37 °C in a humidified incubator with 5% CO_2_ to ensure optimal growth conditions. For the experiments, sub-confluent monolayers were established in 96-well plates and grown in 2% FBS medium. A pseudo-SARS-CoV-2 viral reporter, tagged with fluorescence, was utilized for safety and to mimic viral entry mechanisms. This construct was sourced from Montana Molecular (Bozeman, MT, USA).

### 2.2. Pseudovirus Transduction and Adsorption-Inhibition Assay

The experimental workflow was adapted and expanded from previously published methods by Fuochi et al. [8].

A549 cells (1.5 × 10^5^ cells/well) were plated in 96-well plates and allowed to adhere overnight. To enhance viral entry, the cells were transduced with a red fluorescent ACE2 construct using a BacMam system. Briefly, a red fluorescent transduction mixture containing ACE2 (Santaka-tagged), complete medium, and sodium butyrate was prepared following the supplier’s protocol (Montana Molecular, Live Cell Fluorescent Biosensors). A volume of 50 μL of the mixture, composed of 5 μL ACE2 BacMam (6.6 × 10^8^ VG/mL), 0.6 μL sodium butyrate (2.0 mM final concentration), and 44.4 μL of complete media, was added to each well, and the plate was gently agitated 5–10 times to ensure even distribution. The cells were then incubated again at 37 °C for an additional overnight period. Subsequently, transduction efficiency was assessed using a Leica DM IL LED microscope (© 2023 Leica Microsystems, Buccinasco, 20090, Milan, Italy). Following transduction, the medium was removed and replaced with fresh medium containing HS or EX at concentrations ranging from 8 µg/mL to 1.25 mg/mL. The cells were then treated with a green fluorescent transduction mixture according to the manufacturer’s protocol (Montana Molecular, Fluorescent Biosensors for Live Cell Discovery), which included the neon-green-labeled pseudovirus, fresh complete medium, and sodium butyrate. The preparation of the pseudoviral mixture was obtained following the same previously validated protocol, consisting of 2.5 μL pseudovirus (3.3 × 10^8^ VG/mL), 0.6 μL sodium butyrate (2.0 mM), and GAGs at the indicated concentration range.

Subsequently, this model was used to evaluate the impact of GAGs on viral entry under four different conditions:

- **Treatment A (cell pre-treatment)**: The cells were treated with GAGs for 2 h prior to viral exposure to assess any prophylactic effects.
- **Treatment B (pre-treatment of virus with substances)**: GAGs (individually) and the pseudovirus were co-incubated at 37 °C for 2 h in a water bath. After this pre-incubation, the HS or EX-virus mixture was directly applied to the cells to evaluate the effects of the interaction between the pseudovirus and GAGs during the pre-treatment. This approach aimed to determine how this interaction influences viral infectivity and the cellular response.
- **Treatment C (pre-treatment of virus)**: The pseudovirus was incubated at 37 °C for 2 h in a water bath alone to evaluate the survival of the virus in this condition (internal control of Treatment B). Then, GAGs and the pre-treated pseudovirus were applied to the cells.
- **Treatment D (co-treatment)**: The cells were exposed to the pseudovirus and GAGs simultaneously.

Following the treatment of the cells and pseudovirus as outlined in the experimental protocol, the plate was gently agitated 5–10 times to ensure even distribution of both compounds and viral particles across all wells. The plate was then placed in a standard incubator set at 37 °C with 5% CO_2_ and high humidity. After an incubation period of 12–24 h, the extent of viral adsorption inhibition was evaluated. To remove any unbound virus and residual treatment compounds, the cell monolayers in all experimental conditions were washed twice with pre-warmed phosphate-buffered saline (PBS). A similar washing procedure was applied to the wells where the virus had been pre-incubated with the substances, ensuring that only virus particles capable of successful cell binding during the treatment window were retained for further analysis. This washing step was critical for accurately assessing infectivity by excluding any unbound virus or excess compound that might skew the results. Untreated A549 cells were included as a negative control, providing a baseline measure of viral infectivity in the absence of intervention. This control enabled comparison with the treated groups to determine the inhibitory effects of HS and EX. To control any vehicle-related effects, wells containing only medium and sodium butyrate without GAGs or the pseudovirus were also included. These solution-only controls confirmed that no measurable changes in infectivity or cytotoxicity arose from the vehicle components alone. For clarity and to maintain figure readability, these control data were not shown.

**Figure 1:**
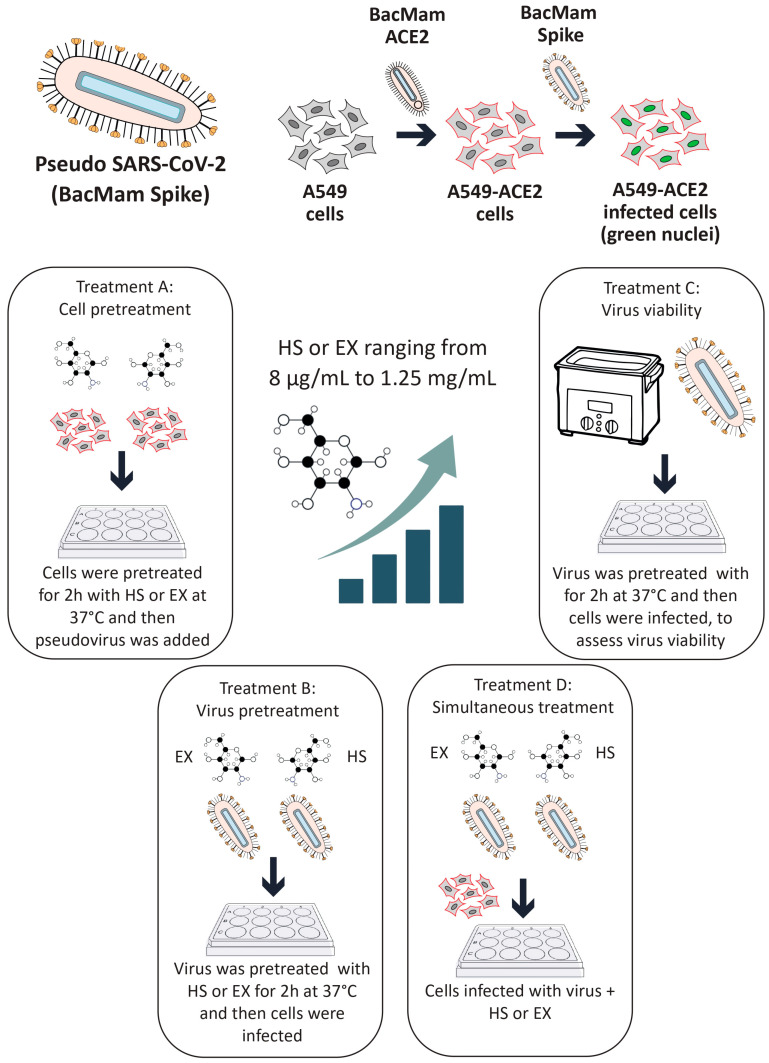
Schematic representation of the experimental setup used to evaluate the antiviral activity of HS and EX against SARS-CoV-2. A549 lung epithelial cells were first transduced with a BacMam ACE2 construct to allow efficient viral entry. The resulting A549-ACE2 cells were then infected with a fluorescently labeled pseudotyped SARS-CoV-2 (BacMam Spike). The antiviral effects of HS and EX (8 μg/mL to 1.25 mg/mL) were assessed under four experimental conditions.

All experimental conditions were tested in quadruplicate to ensure data reproducibility and statistical reliability. This replication strategy helped reduce variability and strengthened the validity of the findings. Transduction efficiency was assessed using a Leica DM IL LED fluorescence microscope in combination with LAS X Life Science software v.1.1.0.12420 (Leica Microsystems, Buccinasco, 20090, Milan, Italy). Semi-quantitative analysis was performed by capturing five representative fields per well. The proportion of transduced cells was estimated using the software’s cell counting module based on fluorescence intensity thresholds: red fluorescence indicated ACE2 membrane expression, while green nuclear fluorescence identified pseudovirus-infected cells. All experiments were independently repeated three times. The entire experimental procedure is described in detail in Figure 1.

### 2.3. Statistical Analyses

All experiments were performed thrice, and data were summarized using the mean (±SD). Where applicable, data were analyzed using two-way ANOVA followed by Tukey’s multiple comparisons test to evaluate the effects of treatment type and concentration. Graphs were generated using GraphPad^®^ Prism ver. 8.4.2.0 (Graph-Pad Software, Boston, MA, USA). A *p* value ≤ 0.05 was considered significant.

## 3. Results

### 3.1. Effects of HS and EX on SARS-CoV-2 Infection

Although direct cytotoxicity assays were not included in the present study, previous work by Fuochi et al. [8] using MTT analysis on A549 cells demonstrated that both HS and EX were well tolerated at concentrations relevant to antiviral activity. Specifically, HS maintained >98% cell viability at 5.0 mg/mL, while EX showed minimal cytotoxic effects, preserving ~87% viability at 1.25 mg/mL. These findings support the safety of the concentration ranges employed in the present study, which remained well below the cytotoxic thresholds previously identified, thereby justifying their use for evaluating antiviral efficacy under physiologically relevant conditions.

#### 3.1.1. Pre-Treatment of Cells (Treatment A)

**Figure 2:**
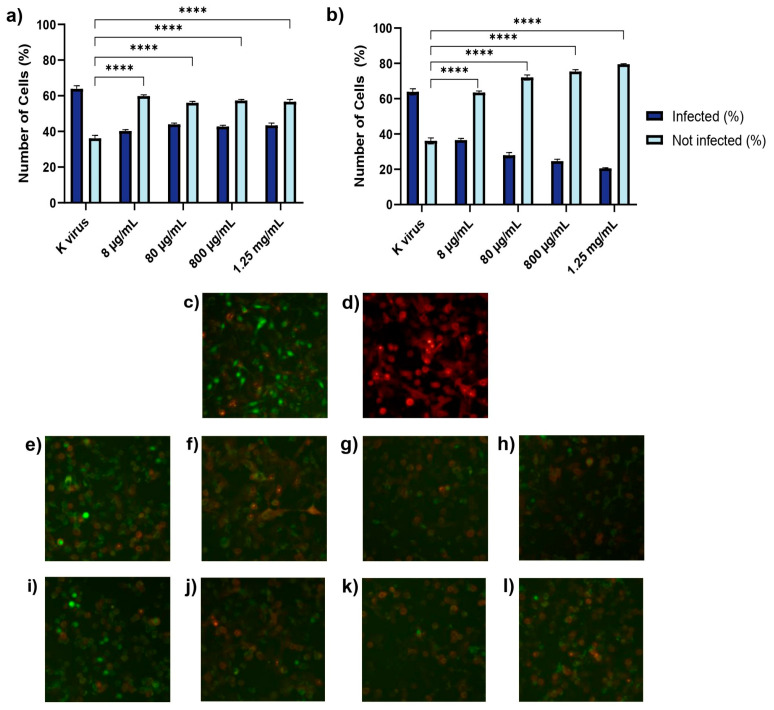
Treatment A: A549 cells were treated with GAGs for 2 h prior to viral exposure to assess any prophylactic effects. (**a**) Histogram displaying the percentage of infected cells following pretreatment with HS. “K virus” indicates cells not pretreated with HS, serving as the control for infection. (**b**) Histogram displaying the percentage of infected cells following pretreatment with EX. “K virus” indicates cells not pretreated with EX, serving as the control for infection. (**c**) Fluorescent micrograph of cells treated with the pseudovirus (K virus): green nuclei indicated infected cells. (**d**) Fluorescent micrograph of ACE2-expressing cells: red fluorescence highlights the expression of ACE2 receptor on the cellular surface. (**e**–**h**) Fluorescent micrographs of A549 cells treated with increasing concentrations of HS: (**e**) 8 μg/mL, (**f**) 80 μg/mL, (**g**) 800 μg/mL, and (**h**) 1.25 mg/mL. (**i**–**l**) Fluorescent micrographs of A549 cells treated with increasing concentrations of EX: (**i**) 8 μg/mL, (**j**) 80 μg/mL, (**k**) 800 μg/mL, and (**l**) 1.25 mg/mL; Images were acquired at 10× magnification. The images illustrate the impact of GAGs on pseudovirus infection rates. Statistical significance was assessed using two-way ANOVA with Tukey’s post hoc test (**** *p*-value < 0.0001).

As shown in Figure 2, pre-treatment of A549 cell GAGs significantly reduced the pseudovirus infection compared to the control group (K virus). For HS, a reduction in infection was observed across all concentrations tested, with infection rates of 37.08% at 8 μg/mL, 31.25% at 80 μg/mL, 33.16% at 800 μg/mL, and 32.09% at 1.25 mg/mL. However, these reductions were not dose-dependent, as the percentages were not statistically different from one another, although each concentration demonstrated significant inhibition compared to the control. In contrast, EX exhibited a clear dose-dependent effect, with infection reduction progressively increasing as the concentration of EX rose—42.76% at 8 μg/mL, 56.19% at 80 μg/mL, 61.43% at 800 μg/mL—and reaching a maximum reduction of 67.91% at 1.25 mg/mL. Fluorescent microscopy corroborated these findings, revealing a consistent decrease in green-stained nuclei (indicating infected cells) with increasing EX concentrations.

#### 3.1.2. Pre-Treatment of the Virus (Treatment B)

**Figure 3:**
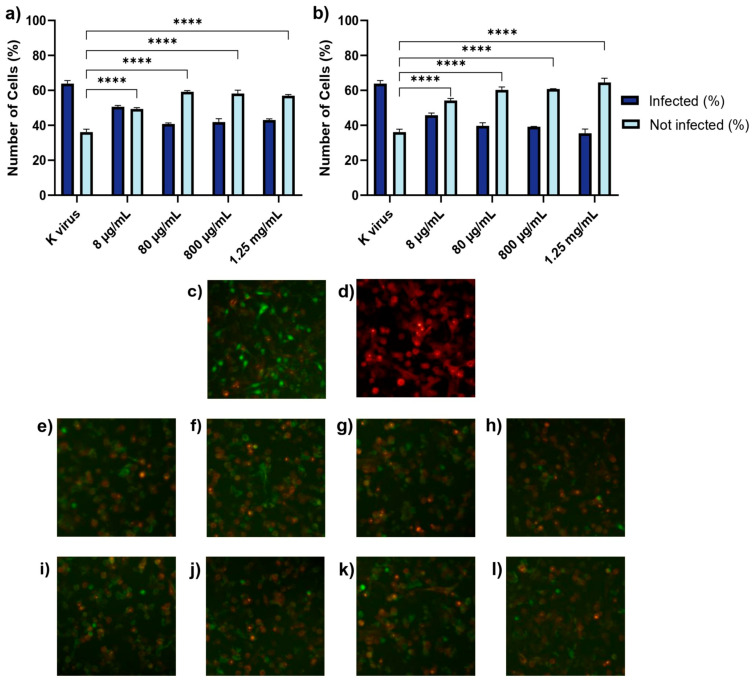
Treatment B: GAGs (individually) and the pseudovirus were co-incubated at 37 °C for 2 h in a water bath. (**a**) Histogram showing the percentage of infected cells after treatment with HS. (**b**) Histogram showing the results of the same treatment using EX. (**c**) Fluorescent micrograph of cells treated with the pseudovirus (K virus): green nuclei indicate infected cells. (**d**) Fluorescent micrograph of ACE2-expressing cells: red fluorescence highlights the expression of ACE2 receptor on the cellular surface. (**e**–**h**) Fluorescent micrographs of A549 cells treated with increasing concentrations of HS: (**e**) 8 μg/mL, (**f**) 80 μg/mL, (**g**) 800 μg/mL, and (**h**) 1.25 mg/mL. (**i**–**l**) Fluorescent micrographs of A549 cells treated with increasing concentrations of EX: (**i**) 8 μg/mL, (**j**) 80 μg/mL, (**k**) 800 μg/mL, and (**l**) 1.25 mg/mL; Images were acquired at 10× magnification. The images illustrate the impact of GAGs on pseudovirus infection rates. Statistical significance was assessed using two-way ANOVA with Tukey’s post hoc test (**** *p*-value < 0.0001).

Pre-treatment of the pseudovirus with GAGs resulted in a significant reduction in infection rates in A549 cells compared to the control group, highlighting the effectiveness of this treatment strategy in mitigating viral infection (Figure 3). For HS, the inhibition of infection was evident at all concentrations, with reductions of 20.78% at 8 μg/mL, 36.24% at 80 μg/mL, 34.55% at 800 μg/mL, and 32.69% at 1.25 mg/mL. However, the reductions were not dose-dependent, in a manner comparable to Treatment A. In contrast, EX exhibited a clear dose-dependent effect, with infection reductions increasing progressively from 28.29% at 8 μg/mL to 44.53% at 1.25 mg/mL. These findings highlight the potential of HS and EX to interfere directly with viral particles, possibly by blocking spike–protein interactions critical for ACE2 receptor binding.

#### 3.1.3. Control Experiment (Treatment C)

**Figure 4:**
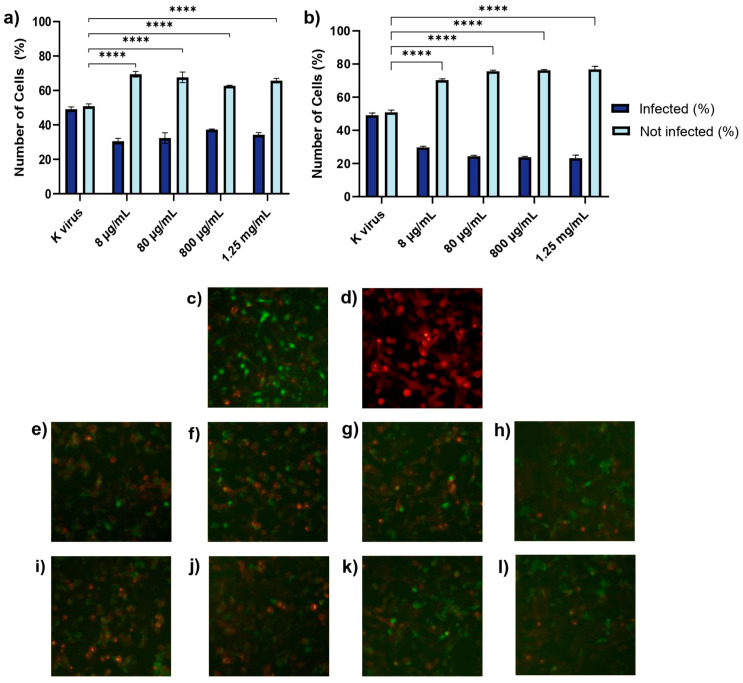
Treatment C: The pseudovirus was pre-treated at 37 °C for 2 h in a water bath to evaluate the survival of the virus in this condition (internal control of Treatment B). Then, GAGs and the pre-treated pseudovirus were applied to the cells. (**a**) Histogram showing the percentage of infected cells after treatment with HS. (**b**) Histogram showing the results of the same treatment using EX. (**c**) Fluorescent micrograph of cells treated with the pseudovirus (K virus): green nuclei indicate infected cells. (**d**) Fluorescent micrograph of ACE2-expressing cells: red fluorescence highlights the expression of ACE2 receptor on the cellular surface. (**e**–**h**) Fluorescent micrographs of A549 cells treated with increasing concentrations of HS: (**e**) 8 μg/mL, (**f**) 80 μg/mL, (**g**) 800 μg/mL, and (**h**) 1.25 mg/mL. (**i**–**l**) Fluorescent micrographs of A549 cells treated with increasing concentrations of EX: (**i**) 8 μg/mL, (**j**) 80 μg/mL, (**k**) 800 μg/mL, and (**l**) 1.25 mg/mL; Images were acquired at 10× magnification. The images illustrate the impact of GAGs on pseudovirus infection rates. Statistical significance was assessed using two-way ANOVA with Tukey’s post hoc test (**** *p*-value < 0.0001).

Pre-incubation of the pseudovirus in the absence of GAGs served as an internal control, confirming that the pseudovirus remained viable and infectious after the preliminary treatment. Approximately 50% of the cells were infected, a result comparable to that of the K virus control. Subsequent treatments with GAGs yielded similar outcomes to those observed in Treatments B and C, demonstrating consistent antiviral effects across these experimental conditions (Figure 4).

#### 3.1.4. Co-Treatment of HS or EX Plus Pseudovirus (Treatment D)

**Figure 5:**
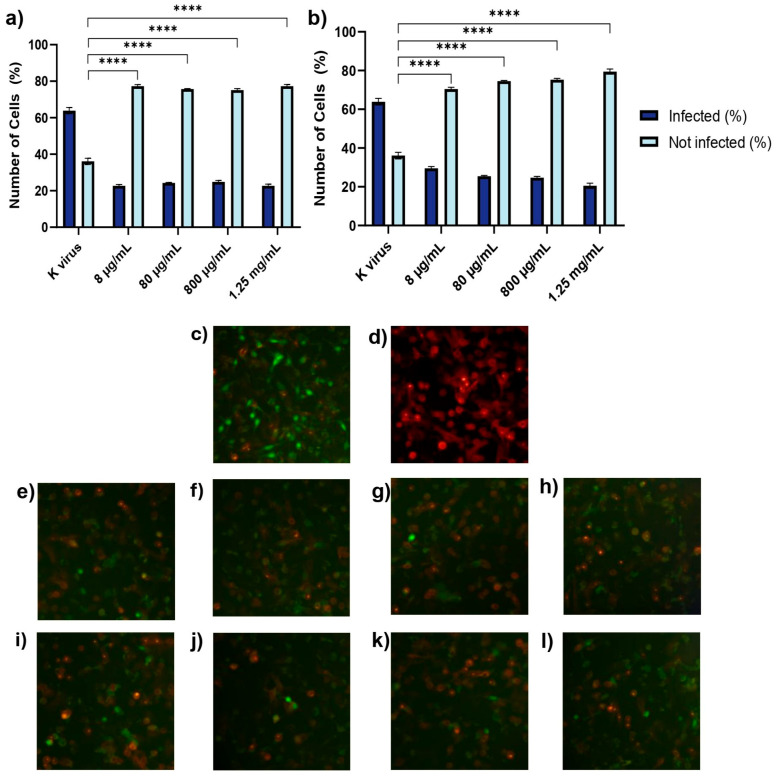
Treatment D: The cells were exposed to the pseudovirus and GAGs simultaneously (co-treatment). (**a**) Histogram showing the percentage of infected cells after treatment with HS. (**b**) Histogram showing the results of the same treatment using EX. (**c**) Fluorescent micrograph of cells treated with the pseudovirus (K virus): green nuclei indicate infected cells. (**d**) Fluorescent micrograph of ACE2-expressing cells: red fluorescence highlights the expression of ACE2 receptor on the cellular surface. (**e**–**h**) Fluorescent micrographs of A549 cells treated with increasing concentrations of HS: (**e**) 8 μg/mL, (**f**) 80 μg/mL, (**g**) 800 μg/mL, and (**h**) 1.25 mg/mL. (**i**–**l**) Fluorescent micrographs of A549 cells treated with increasing concentrations of EX: (**i**) 8 μg/mL, (**j**) 80 μg/mL, (**k**) 800 μg/mL, and (**l**) 1.25 mg/mL; Images were acquired at 10× magnification. The images illustrate the impact of GAGs on pseudovirus infection rates. Statistical significance was assessed using two-way ANOVA with Tukey’s post hoc test (**** *p*-value < 0.0001), scale bar.

When A549 cells were simultaneously exposed to the pseudovirus and either HS or EX, infection rates declined significantly compared to the untreated controls (Figure 5). At equal concentrations, HS demonstrated a superior inhibitory effect compared to EX. Specifically, at a lower concentration of 8 μg/mL, HS achieved an inhibitory effect of 64.86%, whereas EX displayed a lower inhibitory effect of 53.78%. For HS, the inhibitory effects at other concentrations were 61.95% at 80 μg/mL, 61.12% at 800 μg/mL, and 64.43% at 1.25 mg/mL. In contrast, the inhibitory effects for EX were 60.10% at 80 μg/mL, 61.30% at 800 μg/mL, and 67.84% at 1.25 mg/mL. Overall, the inhibitory effect of HS was not dose-dependent but remained consistently more potent than that of EX. In contrast, treatment with EX exhibited a clear dose-dependent effect.

## 4. Discussion

The constant antiviral activity of HS across all tested concentrations may reflect its ability to act as a competitive binder, saturating spike-accessible glycocalyx domains without requiring high-affinity interactions. In contrast, the progressive inhibition seen with EX supports a more targeted interaction with the spike protein, possibly involving specific sulfation patterns or conformational constraints. These differences may be especially relevant in the context of emerging SARS-CoV-2 variants, where GAG-binding domains tend to be more conserved than ACE2-contacting residues. Unlike monoclonal antibodies or protein-based inhibitors, which typically target variable regions of the spike protein and require systemic administration, glycosaminoglycans such as HS and EX interact with more conserved binding domains, offering a sequence-independent antiviral approach potentially compatible with topical or intranasal delivery [2,5]. Therefore, the distinct profiles of HS and EX might offer complementary coverage in future therapeutic formulations, especially in combination or layered delivery strategies. This study demonstrated the differential efficacy and potential mechanisms of action of HS and EX in inhibiting SARS-CoV-2 spike protein interactions under various experimental conditions.

The rationale for selecting HS and EX stems from their distinct yet complementary biological properties. HS, as a naturally occurring component of the host cell glycocalyx, facilitates initial contact between SARS-CoV-2 and the cell surface, thereby contributing to the early stages of viral adhesion and concentration at the membrane level. In contrast, EX is a low-molecular-weight heparin widely used in clinical practice, with an established safety profile and regulatory approval for systemic administration. Importantly, EX has been investigated for pulmonary and intranasal delivery, with preclinical studies showing favorable tolerability, local retention, and minimal systemic absorption, making it a viable candidate for respiratory antiviral interventions [10,11]. These features highlight the potential of EX to serve as a dual-action agent, simultaneously interfering with viral entry and modulating inflammation within the respiratory mucosa. Moreover, to clarify the reason behind each experimental condition, the three treatment strategies were designed to simulate distinct therapeutic scenarios relevant to clinical application. Pre-treatment of host cells (Treatment A) was intended to model a prophylactic intervention, reflecting a preventive approach where host tissues are pre-exposed to GAGs, as would occur with a nasal spray or inhaled formulation prior to viral contact. Pre-treatment of the viral particles (Treatment B) explored the direct antiviral potential of HS and EX through their interaction with the virus before cell entry, mimicking extracellular neutralization. Lastly, simultaneous exposure (Treatment D) simulated a real-time therapeutic scenario, where antiviral agents are present during viral exposure, offering insight into both preventive and early treatment contexts. These distinct approaches help delineate the timing, mechanism, and clinical relevance of HS and EX administration in SARS-CoV-2 infection management.

These findings align with previous evidence that cell surface HS acts as a co-receptor for SARS-CoV-2 [4] and that exogenous sulfated polysaccharides can neutralize pseudovirus particles [12]. Furthermore, binding studies confirm that EX directly interacts with spike RBD and modulates protease activity, supporting its multifunctional antiviral capacity [13]. Co-treatment data integrate these mechanisms, suggesting combined prophylactic and early therapeutic applicability.

These findings underscored the distinct roles of each compound and provided important insights into their antiviral mechanisms. The antiviral effects of HS and EX varied across the three experimental treatment modalities, reflecting their distinct mechanisms of action. As shown in Treatment A (cell pre-treatment), HS significantly inhibited viral infection at all tested concentrations, but its effect plateaued, suggesting that once critical viral binding sites were blocked, further increases in concentration did not enhance efficacy. This behavior was likely due to HS mimicking the natural GAG layer of the cellular glycocalyx, sequestering viral particles and preventing their interaction with the cell surface. In contrast, EX exhibited a clear dose-dependent effect, with higher concentrations progressively reducing viral entry. This suggests that EX acted through direct binding to the spike protein, impairing its ability to establish productive interactions with the host cell. The mimicking role of HS and its competition with viral binding sites has been supported in previous studies [14]. The dose-dependent nature of EX aligns with findings from structural studies on GAGs and their effects on viral proteins. When the pseudovirus was pre-incubated with HS or EX (Treatment B), both compounds substantially reduced infectivity. HS’s consistent inhibition across concentrations likely resulted from a steric hindrance, where HS molecules masked the spike protein’s binding regions, reducing its capacity to interact with host cells. EX’s dose-dependent inhibition indicated that its increasing concentration enhanced the likelihood of multi-site binding on the spike protein, thereby reducing the virus’s ability to interact effectively with its cellular receptor. The concept of steric hindrance as a potential mechanism for HS aligns with the experimental findings in GAG-mediated viral neutralization. Similarly, EX’s interactions with the spike protein have been suggested to involve multi-site binding and conformational changes, enhancing its inhibitory effects [15]. Finally, simultaneous exposure to GAGs and the pseudovirus revealed nuanced differences (Treatment D). HS showed superior inhibition at lower concentrations compared to EX, highlighting its ability to interfere early in the viral attachment process, likely by outcompeting the virus for interactions with cellular heparan sulfate proteoglycans. However, at higher concentrations, EX achieved comparable or superior inhibition, suggesting that its progressive saturation of spike protein binding sites eventually blocked viral entry as effectively as HS. These results highlight the complementary nature of HS and EX in inhibiting SARS-CoV-2 infection. This duality opens up the possibility for synergistic strategies combining viral decoys and direct inhibitors, as for other receptors [16]. While HS primarily acted as a competitive decoy, EX’s dose-dependent activity suggested strong interactions with viral components, making both compounds valuable therapeutic candidates. Experimental studies have also highlighted the competitive nature of HS in similar contexts [14]. The progressive inhibition of EX with higher doses aligns with its molecular properties and its use as a low-molecular-weight heparin [7]. Although ACE2 is the primary receptor mediating SARS-CoV-2 entry, alternative pathways involving Toll-like receptors TMPRSS2 and SR-B1 have also been implicated. Thus, it is plausible that HS and EX might exert additional inhibitory effects by interfering with these auxiliary receptors, a hypothesis warranting further investigation. Though the present experiments focused on pseudoviruses expressing the wild-type spike protein, previous molecular modeling studies conducted by Fuochi et al. demonstrated that both HS and EX retained strong binding affinity to the receptor-binding domains of several SARS-CoV-2 variants, including BA.2.86, KP.2, and JN.1. These variants exhibited extensive spike mutations, yet docking analysis confirmed the conservation of key interaction residues, most notably Gly496 and Gln498, across all tested isoforms [8]. These results suggest a broad-spectrum inhibitory potential of HS and EX, which might have been preserved despite spike protein evolution.

Additionally, GAGs such as HS have been shown to influence TLR-mediated inflammation, suggesting possible anti-inflammatory contributions [17]. Moreover, HS and EX appeared to exert their antiviral effects primarily through interactions with the spike protein rather than directly targeting ACE2. Electrostatic interactions between the negatively charged sulfate groups of HS and EX and the positively charged residues on the RBD of the spike protein likely mediated these effects. For HS, these interactions mimicked natural viral binding to the glycocalyx, diverting viral particles from cellular targets. EX, with its lower molecular weight and specific sulfation pattern, possibly induced conformational changes in the spike protein upon binding, impairing its ability to interact with ACE2. Virus pre-treatment provided evidence for steric hindrance. By occupying key binding regions on the spike protein, HS and EX likely created physical barriers that prevented the virus from approaching the cell surface closely enough to engage productively. This mechanism was particularly evident for HS, which showed consistent inhibition across all concentrations, suggesting that steric effects saturated once the viral surface was sufficiently masked. The dose-dependent action of EX likely resulted from its ability to bind progressively to the spike protein, creating cumulative disruptions in viral attachment and fusion. Higher concentrations likely increased the probability of multi-site binding, enhancing inhibitory potential. In contrast, HS’s consistent efficacy across concentrations reflected a saturable mechanism: once the viral surface was sufficiently covered, additional HS molecules provided no further benefit. These distinct behaviors highlighted the structural and functional differences between the two GAGs. The electrostatic interactions of HS and EX with spike proteins, including conformational changes upon binding, have been explored in molecular studies [1,18,19].

This study introduced a significant level of novelty compared to previous investigations [20], as the aim was to develop a formulation suitable for nasal administration. To this end, lower concentrations of both HS and EX were tested [8,10], while rigorously assessing their antiviral efficacy under distinct treatment conditions (cell pre-treatment, virus pre-treatment, and co-treatment). This approach is bolstered by preliminary studies that used air–liquid interface (ALI) exposure to assess the cytotoxicity of aerosols generated from nebulized solutions of these compounds [8,14]. However, those studies predominantly focused on higher dosages. By intentionally lowering the tested doses, excellent inhibition of viral entry was achieved, thereby demonstrating that effective antiviral activity can be maintained. The combination of a lower-dose approach and the use of different treatment strategies not only highlights the different ways HS and EX work, but also shows the potential to develop safer, targeted antiviral treatments with nasal administration. This could help overcome some of the limitations seen in earlier studies. This innovation, which integrates optimized dosing with a delivery method designed for direct application to the respiratory tract, represents a significant advancement over the existing literature and provides a promising foundation for the development of next-generation prophylactic or therapeutic strategies against SARS-CoV-2 [11].

Although the pseudovirus model enables a safe and controlled assessment of viral entry inhibition, it does not capture the full complexity of SARS-CoV-2 infection, including post-entry replication, immune evasion mechanisms, and systemic effects [21,22].

Moreover, given the in vitro nature of the current study, in vivo evaluations are essential to assess pharmacodynamics, biodistribution, mucosal retention, and immune interactions under physiological conditions. Therefore, future studies using live viruses or in vivo models will be required to fully validate these findings.

## 5. Conclusions

This study demonstrated that HS and EX effectively inhibit SARS-CoV-2 pseudoviral entry through distinct yet complementary mechanisms. HS acted as a competitive decoy at the host cell surface, mimicking endogenous glycosaminoglycans and sterically hindering spike engagement, while EX exerted dose-dependent interference through direct binding to the viral spike protein. This dual strategy supports the use of GAG-based inhibitors targeting early infection events through conserved structural domains, independent of viral sequence variability.

Importantly, the results revealed that HS maintains inhibitory capacity across all tested concentrations, suggesting a saturation threshold, whereas EX displays a linear dose–response profile, indicating its adjustable antiviral potency. These data support the application of both compounds—either alone or in combination—for intranasal or topical administration in preventive or early therapeutic contexts.

This study clarified how the distinct molecular interactions between HS, EX, and the spike protein contribute to their antiviral activity, offering a rationale for the design of targeted therapeutic interventions. Given the favorable safety profiles and suitability for local delivery, HS and EX represent promising candidates for translational development, particularly in scenarios where viral antigenic drift compromises vaccine or antibody efficacy. This hypothesis is further supported by recent findings showing that intranasal heparin administration significantly inhibits SARS-CoV-2 replication in human nasal epithelial cells, providing strain-agnostic protection at the primary site of viral entry [23].

However, the in vitro pseudoviral system employed does not fully replicate the complexity of live virus infection, including post-entry replication, systemic dissemination, and immune modulation.

Further investigations using infectious viral models and in vivo systems are required to evaluate pharmacokinetics, mucosal bioavailability, and therapeutic potential under physiological conditions. Additionally, the potential anti-inflammatory effects of HS and EX on the respiratory tract remain to be elucidated.

In summary, the findings establish a scientific basis for advancing GAG-mimetic antiviral strategies, emphasizing their utility as broad-spectrum, non-sequence-dependent tools for the management of SARS-CoV-2 and related respiratory pathogens.

## Data Availability

The original contributions presented in this study are included in the article. Further inquiries can be directed to the corresponding authors.

## Abbreviations

The following abbreviations are used in this manuscript:

ACE2	Angiotensin-Converting Enzyme 2
EX	Enoxaparin
GAGs	Glycosaminoglycans
HS	Heparan Sulfate
SARS-CoV-2	Severe Acute Respiratory Syndrome Coronavirus 2
